# Correspondence

**Published:** 1868-07

**Authors:** 


### CORRESPONDENCE.

PROF. TAFT.—*Dear Sir :* In one of your lectures, session before last, you stated that you occasionally found a nerve of a tooth whose vitality was not overcome even by repeated applications of arsenious acid. Having noticed the same thing, before and since, myself, I was led to give some attention to the reason of it.

From my investigations, I have come to the following conclusions. All freshly exposed and abraded pulps, having the mouths of the capillaries opened, whereby the arsenious acid is taken directly in (or if, as some contend, that it is not taken into the circulation) it acts dynamically, by being brought into sensible contact with the vital part, and the life is thereby destroyed. Whereas, pulps that have been exposed for a long time, the reparative process is established, which produces a granular surface, exuding new cells, serum or pus, and it may be elaborating ossific matter (secondary dentine). Now these form a barrier to the applied devitalizer. Instead of it being taken up or absorbed into the pulp, it is excluded or thrown away from the vital part by this continual exudation. This also prevents catalytic action, because the devitalizer is at a sensible distance from the vital part, and is kept so by the barrier before alluded to.

Consequently, no injury is done to the vitality of the pulp by the application of arsenious acid when this condition of the pulp exists.

Some one may ask, why is it that arsenious acid will devitalize a pulp through a wall of dentine of considerable thickness? The reason is obvious, there are hundreds of nerve fibrils passing through the dentine.

Now, if arsenious acid is placed in contact with the exposed ends of these nerve fibrils, in the cavity of decay, and they are in a condition to receive the devitalizing effect of the acid, it will be conveyed to the pulp, and death to it will ensue.

Now, if these conclusions are correct, what must be done to a pulp that refuses to give up its vitality? The answer is remove the granular surface down to the vital part and apply the devitalizer in the usual way.

Very Respectfully,

M. McCARTY.

